# Visual-to-Tactile Cross-Modal Generation Using a Class-Conditional GAN with Multi-Scale Discriminator and Hybrid Loss

**DOI:** 10.3390/s26020426

**Published:** 2026-01-09

**Authors:** Nikolay Neshov, Krasimir Tonchev, Agata Manolova, Radostina Petkova, Ivaylo Bozhilov

**Affiliations:** Faculty of Telecommunications, Technical University of Sofia, 8 Kliment Ohridski Blvd., 1000 Sofia, Bulgaria; k_tonchev@tu-sofia.bg (K.T.); amanolova@tu-sofia.bg (A.M.); rapetkova@tu-sofia.bg (R.P.); ibojilov@tu-sofia.bg (I.B.)

**Keywords:** augmented reality, conditional Generative Adversarial Network (cGAN), cross-modal generation, haptic feedback, hybrid loss, LMT-108 dataset, multi-scale discriminator, texture-to-tactile translation, vibrotactile synthesis, virtual reality

## Abstract

Understanding surface textures through visual cues is crucial for applications in haptic rendering and virtual reality. However, accurately translating visual information into tactile feedback remains a challenging problem. To address this challenge, this paper presents a class-conditional Generative Adversarial Network (cGAN) for cross-modal translation from texture images to vibrotactile spectrograms, using samples from the LMT-108 dataset. The generator is adapted from pix2pix and enhanced with Conditional Batch Normalization (CBN) at the bottleneck to incorporate texture class semantics. A dedicated label predictor, based on a DenseNet-201 and trained separately prior to cGAN training, provides the conditioning label. The discriminator is derived from pix2pixHD and uses a multi-scale architecture with three discriminators, each comprising three downsampling layers. A grid search over multi-scale discriminator configurations shows that this setup yields optimal perceptual similarity measured by Learned Perceptual Image Patch Similarity (LPIPS). The generator is trained using a hybrid loss that combines adversarial, $L_{1}$, and feature matching losses derived from intermediate discriminator features, while the discriminators are trained using standard adversarial loss. Quantitative evaluation with LPIPS and Fréchet Inception Distance (FID) confirms superior similarity to real spectrograms. GradCAM visualizations highlight the benefit of class conditioning. The proposed model outperforms pix2pix, pix2pixHD, Residue-Fusion GAN, and several ablated versions. The generated spectrograms can be converted into vibrotactile signals using the Griffin–Lim algorithm, enabling applications in haptic feedback and virtual material simulation.

## 1. Introduction

Immersive virtual environments, including both Virtual Reality (VR) and Augmented Reality (AR) systems, seek to enhance user experience through rich multisensory interactions. Although both VR and AR systems primarily rely on visual and auditory feedback, these modalities often fall short in conveying the tactile sensations essential for realism. Tactile feedback technologies have broad potential applications in areas such as remote manipulation [1], virtual training [2], medical simulation [3], real-time user–avatar environments [4], and robotics [5]. In particular, AR scenarios can benefit from tactile feedback aligned with visual overlays, enabling more natural interaction with virtual content superimposed on real-world surfaces. However, generating realistic tactile sensations with touch sensors can be inconvenient, especially for real-time applications, and the high cost of tactile hardware further motivates research into vision-based approaches for touch-related tasks. Methods that generate vibrotactile data directly from visual sources, such as cameras, are therefore of growing interest [6,7,8,9,10,11,12,13]. This idea mirrors the human capacity for cross-modal perception, where visual cues often allow intuitive inference of tactile properties like smoothness or roughness without physical contact. In robotics, the ability to infer tactile properties from vision alone can support object manipulation, material recognition, and safe human–robot interaction, especially in cases where tactile sensors are unavailable or impractical. Similar challenges arise in immersive environments, where visual information must often compensate for the absence of direct touch. Hence, vibrotactile feedback, which conveys surface texture information, plays a crucial role in enhancing the sense of presence. Moreover, such vision-driven vibrotactile generation could support formation control in multi-agent systems or autonomous vehicles [14,15], where haptic cues enhance coordination, obstacle negotiations, and safe interactions among agents.

Within this context, two major research directions have emerged: generating vibrotactile signals from visual images [6], and translating between visual and GelSight-based tactile images [7,8,9,10,11,12,13]. Vibrotactile signals, unlike tactile data from GelSight-based images, are inherently one-dimensional time-domain data, differing substantially from the two-dimensional structure of images, making standard image-to-image translation frameworks unsuitable for direct application. To overcome this challenge, most existing approaches transform time-domain vibrotactile signals into time-frequency representations (spectrograms), which are essentially treated as images, making them compatible with powerful image-based generative models such as Generative Adversarial Networks (GANs) [16]. It should be noted that the difference between surface images and spectrogram images is considerably greater in both spatial and temporal characteristics than the difference between surface images and GelSight-based tactile images, which makes generating spectrograms a more challenging task.

To address this, researchers have leveraged various GAN-based architectures originally developed for image-to-image translation and cross-modal generation [17]. In particular, conditional Generative Adversarial Network (cGAN), such as pix2pix [18] and its high-resolution extension pix2pixHD [19], is widely used in paired settings, where the source and target domains are aligned. These models condition the generation process on the input image, allowing the discriminator to receive both the generated image and the input image, which helps in producing more controlled and consistent outputs. On the other hand, models like CycleGAN [20] and DiscoGAN [21] are designed for unpaired domain translation, where the correspondence between source and target samples is unknown. These architectures utilize cycle-consistency constraints to enforce semantic alignment between domains without requiring explicitly paired data. More recent models such as SPatially-Adaptive (DE) normalization (SPADE) [22] introduce spatially-adaptive normalization layers, enabling finer control over the generation process based on semantic layouts or structured inputs. The general principles of both paired and unpaired generative models serve as a foundation for designing effective cross-modal translation pipelines, particularly in tasks involving the translation of visual characteristics to vibrotactile signals.

Building on these foundations, this paper proposes a cGAN-based framework for cross-modal generation, working in a paired data setting. The method follows the principles of the pix2pix and pix2pixHD architectures and introduces label conditioning in the generator to facilitate the translation. Specifically, it focuses on generating spectrogram representations of vibrotactile signals from texture input, which can subsequently be transformed back into time-domain signals using the Griffin–Lim algorithm [23]. This two-stage process enables the realistic rendering of tactile sensations through haptic interfaces. The main contributions of this work are as follows:

- A class-conditional GAN architecture is proposed for generating vibrotactile spectrograms from texture images of material surfaces. The generator is adapted from pix2pix [18], while the discriminator follows the multiscale structure of pix2pixHD [19]. An optimal configuration with three discriminators and three downsampling layers was determined through grid search. Class conditioning is implemented via Conditional Batch Normalization (CBN) [24] at the generator’s bottleneck, based on the material label predicted by a separately trained classifier. The effectiveness of this approach is further illustrated using GradCAM visualizations.
- A hybrid loss function is utilized for training, which combines $L_{1}$ loss, Feature Matching (FM) loss, and adversarial loss components to better guide the generator. This hybrid loss improves both structural accuracy and perceptual quality of the generated spectrograms.
- An extensive evaluation was conducted on samples from 9 materials in the LMT-108 Surface-Materials dataset [25], including qualitative inspection of real vs. generated spectrograms and quantitative assessment using the LPIPS and FID metrics. The results demonstrate superior performance compared to three baseline models (pix2pix, pix2pixHD, and Residue-Fusion GAN), as well as several ablated variants. Ablation studies focus on the impact of removing $L_{1}$ loss, FM loss, and using a single discriminator, highlighting the contribution of each component to the final performance. Each experiment was repeated 10 times to ensure robustness. Two-sided paired *t*-tests comparing the proposed method to each baseline and ablated variant confirmed the statistical significance of the improvements. Representative failure cases are also discussed to highlight remaining challenges.

The rest of the paper is organized as follows: Section 2 reviews related work in the field of cross-modal translation and vibrotactile signal generation. Section 3 details the proposed architecture and its implementation. Section 4 presents the datasets and experimental setup, describes the selection process for the optimal discriminator configuration, and provides both qualitative and quantitative comparisons against baseline models and ablated variants. Additionally, this section illustrates the impact of class-conditional batch normalization through GradCAM [26] visualizations and discusses representative failure cases. Section 5 outlines the limitations of the proposed approach. Finally, Section 6 concludes the paper.

## 2. Related Works

The present study addresses a specific challenge within the domain of cross-modal learning, which explores how information from different sensory modalities can be leveraged or translated. Recent advancements in deep learning, including GANs, Variational Autoencoders (VAEs) [27], and transformers [28], have significantly propelled cross-modal learning, offering robust architectures for generating and translating between modalities. These models benefit from large-scale, diverse datasets that help mitigate domain-specific biases and improve generalization. While significant progress has been made in modality transfers such as text-to-image [29,30], audio-to-visual [31,32], and text-to-speech [29], the specific task of generating vibrotactile signals or their time-frequency representations (e.g., spectrograms) from visual inputs has received comparatively less attention.

Early contributions in this direction are presented by Ujitoko et al. [33] and Ban et al. [34], both of whom utilize a cGAN framework to synthesize spectrograms from visual representations of textured surfaces. In these approaches, the generator is conditioned on a class label, which can be either provided directly or predicted by a separately trained classifier network, along with a latent noise vector to guide the generation process. The methods show promising results in capturing perceptually relevant frequency patterns. However, the use of a cGAN conditioned only on class label, without incorporating the actual texture image, may limit the spatial fidelity of the outputs, especially for more complex textures. In contrast, the method proposed in this work utilizes both the predicted label and the texture image as inputs to the cGAN, allowing for richer and more spatially grounded generation.

In two other works, Cai et al. [35,36] adopted a GAN framework to generate friction signals from texture images of fabric materials. In another study [37], the authors proposed Residue-Fusion GAN, a method for visual-tactile cross-modal data generation using a cGAN architecture combined with a residue-fusion module. This module provides feature representation of label information to the generator, extracted by a pre-trained classifier. Moreover, the authors of [37] propose training the model with additional FM and perceptual losses to facilitate the cross-modal generation process.

Xi et al. [38] recently introduced CM-AVAE, a Cross-Modal Adversarial Variational Autoencoder designed for visual-to-tactile data generation. Their model integrates latent space learning from variational autoencoders into the GAN framework, where the generator’s decoder feature vectors are mapped to the discriminator. This allows for more flexible and informative generation of tactile data from visual inputs, leveraging both the strengths of autoencoders and adversarial training. However, in the last two methods [37,38], VGG-19 [39] features are employed to compute a perceptual component of the reconstruction loss between generated and real spectrograms, which may not be the most appropriate choice, since VGG-19 is pretrained on natural images rather than spectrogram data. Moreover, both methods incorporate a single discriminator. In contrast, the architecture proposed in this work utilizes a hybrid loss that combines $L_{1}$, FM loss, and adversarial loss to train the generator, aiming to improve both structural accuracy and perceptual quality of the generated spectrograms. Moreover, differently from the other described methods, the proposed model incorporates a multi-scale approach with three discriminators, each conditioned on both the generated and real spectrograms at different resolution levels, enabling more effective learning of multi-scale structure and perceptual alignment.

Consistent with previous works [35,37,38,40], evaluating how the reconstructed time-domain vibrotactile signals match the original signals falls beyond the scope of this paper, as the focus is solely on the similarity of the spectrogram patterns. This design choice is motivated by the fact that GAN-based models are particularly well-suited for image-to-image translation tasks, such as spectrogram synthesis, whereas reliable generation of time-domain signals remains an open challenge.

Another class of algorithms employed for various generative modeling tasks is based on diffusion models, including [41,42,43,44,45,46] and transformer-based architectures [47,48]. Some recent studies [49,50,51] have applied diffusion-based approaches primarily for tactile data generation related to GelSight or GelSight-like optical tactile sensors. In these systems, tactile information is acquired as high-resolution images capturing the deformation of a soft elastomer surface under physical contact, effectively treating tactile sensing as an image generation or image translation problem. This sensing and actuation paradigm differs fundamentally from the vibrotactile actuator setting considered in this work. Here, tactile feedback is delivered through actuator-driven vibration signals, which are naturally represented in the time-frequency domain as spectrograms. Unlike optical tactile images, vibrotactile signals emphasize temporal dynamics and frequency content rather than spatial deformation patterns. Consequently, the objectives and data representations in diffusion- or transformer-based optical tactile synthesis are not directly transferable to vibrotactile signal generation. In this context, spectrogram-based GAN architectures remain a practical and effective solution, enabling direct image-to-spectrogram translation. Moreover, diffusion and transformer models typically require larger datasets and greater computational resources, which can pose challenges in the relatively niche domain of visual-to-vibrotactile signal generation. Exploring these approaches for actuator-driven tactile signal synthesis remains a promising avenue for future research.

Among all the approaches considered so far, the architecture proposed in our paper shares the most structural similarities with Residue-Fusion GAN [37]. Both approaches adopt a GAN framework, employ U-Net–style generators (although the two generators differ in their internal design), and incorporate label conditioning driven by a DenseNet-based classifier. However, the way semantic information is injected into the generator differs fundamentally. In Residue-Fusion GAN, classifier-extracted feature maps are fed into the generator through a dedicated Residue Fusion module, creating a feature-level conditioning pathway. In contrast, our method avoids feature-level fusion entirely: the model uses only the predicted class label, which modulates the latent representation within the generator via CBN applied at the bottleneck. This leads to a substantially simpler and more stable conditioning mechanism.

Beyond the conditioning strategy, the discriminator designs are also markedly different. Residue-Fusion GAN relies on a single discriminator, whereas our method adopts a multi-scale discriminator with an optimized number of discriminators and downsampling stages, determined through grid search, yielding improved robustness to texture variability across spatial scales.

Residue-Fusion GAN employs a WGAN-GP (Wasserstein Generative Adversarial Network with Gradient Penalty) as the adversarial component of its training, together with VGG-based perceptual loss and feature matching loss. In contrast, our method follows the adversarial learning paradigm of pix2pix and pix2pixHD, using standard GAN losses across multiple discriminators together with reconstruction loss and feature matching loss. This choice is motivated by the structured, conditional nature of the visual-to-tactile generation task, where stable pixel-level supervision plays a critical role. Moreover, unlike natural images, tactile spectrograms do not align well with the feature space learned by VGG-19, which limits the effectiveness of perceptual losses in this domain. Our experiments confirm that reconstruction loss provides more reliable and task-relevant guidance for tactile spectrogram synthesis, while the multi-scale discriminator setup ensures stable adversarial training without the additional complexity introduced by gradient penalty terms.

A more detailed conceptual, quantitative, and qualitative comparison between Residue-Fusion GAN and our proposed approach is presented in Section 4.3, where we further highlight the improved performance and architectural advantages of our method.

## 3. Proposed Method

### 3.1. Block Diagram

**Figure 1:**
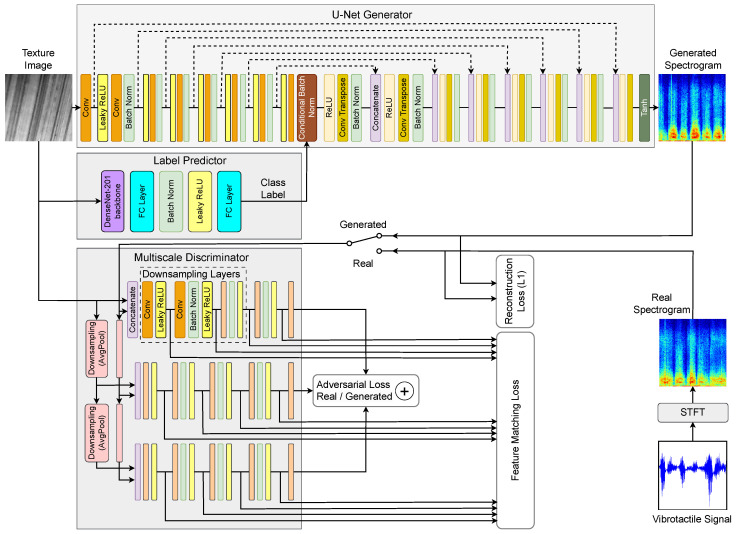
Block diagram of the proposed class-conditional GAN architecture for visual-to-tactile cross-modal generation, where colored functional blocks with descriptions correspond to the other blocks of the same color without descriptions, indicating the same functionality. The generator follows a U-Net encoder-decoder structure with skip connections and integrates CBN at the bottleneck, conditioned on labels predicted by a DenseNet-based classifier. The discriminator employs a multi-scale setup composed of three discriminators, each with three downsampling layers, enabling effective analysis at different resolutions. Training is guided by a hybrid loss that combines adversarial, $L_{1}$, and FM terms to improve perceptual quality.

The architecture of the proposed method is shown in Figure 1 and represents a cGAN. It takes a grayscale Texture Image as input, which is simultaneously fed into both the Generator and the Label Predictor modules. The Label Predictor is a classification network built on a DenseNet-201 backbone [52], trained separately to predict a texture class label from the input image. This predicted label is used to condition the generator through CBN applied at the bottleneck.

The role of the Generator is to synthesize a Generated Spectrogram that represents the tactile characteristics associated with the input texture image. The Real Spectrogram is derived from the ground-truth Vibrotactile Signal by applying a Short-Time Fourier Transform (STFT). The Generator and the Multi-Scale Discriminator are trained jointly in an adversarial setting, following the standard cGAN framework. Training is guided by a hybrid loss function composed of three terms: an adversarial loss, an $L_{1}$ reconstruction loss between real and generated spectrograms, and an FM loss computed from intermediate discriminator features.

The following sections describe each component shown in Figure 1 in more detail.

**U-Net Generator with Class Conditioning:** The generator follows a standard U-Net architecture based on the pix2pix [18] framework with eight downsampling (encoder) and eight upsampling (decoder) layers, as shown in Figure 1. This choice follows the standard pix2pix design for 256 × 256 inputs, where each downsampling step reduces the spatial resolution by a factor of two, resulting in a compact bottleneck representation. Skip connections between corresponding encoder and decoder layers are used to preserve spatial details. Although the original pix2pix model does not include class conditioning, we extend it by incorporating class information into the bottleneck layer. Preliminary experiments with shallower and deeper variants showed that fewer stages lead to insufficient modeling capacity, while additional stages increase computational cost without yielding consistent perceptual improvements.

The choice of the U-Net over the ResNet-based generator in pix2pixHD is motivated by recent works [35,37,38], which highlight the advantage of U-Net-like architectures with skip connections for tasks involving image-to-spectrogram synthesis. The spatial locality and symmetry of U-Net enable the generator to retain low-level structural features, allowing the bottleneck to act as a compact and effective control point for injecting class information, which is implemented using CBN (explained later). In contrast, initial investigations show that the deeper and more abstract representation learned by the ResNet-based encoder in pix2pixHD [19] tends to diffuse spatial cues early, making single-point conditioning less effective. A detailed description of the generator components and their parameters is provided in Table 1.

**Conditional Batch Norm:** A key component of the proposed architecture is the use of CBN [24], applied to the latent feature variable at the bottleneck of the generator. Let $x\in R^{B\times F}$ denote this latent feature variable, where *B* is the batch size and *F* is the feature dimensionality. Let $y\in R^{B\times C}$ represent the one-hot encoded class labels, where *C* is the number of classes.

The CBN layer computes the scale (γ) and shift (β) parameters conditioned on the class labels through learned linear projections:

(1) $$\gamma =yW_{\gamma }+b_{\gamma }\in R^{B\times F},$$

(2) $$\beta =yW_{\beta }+b_{\beta }\in R^{B\times F}.$$

Here, $W_{\gamma }\in R^{C\times F}$ and $W_{\beta }\in R^{C\times F}$ are learnable weight matrices, while $b_{\gamma }\in R^{F}$ and $b_{\beta }\in R^{F}$ are learnable bias vectors broadcast across the batch dimension. Their values are determined automatically during training, and they implement linear projections from the class label space to the feature space.

The normalized latent feature variable $x_{\mathrm{norm},\;y}$ is then computed as:

(3) $$x_{\mathrm{norm},\;y}=\gamma \cdot \frac{x-\mu }{\sigma }+\beta ,$$

where μ and σ are the mean and standard deviation computed across the batch for each feature dimension. This formulation allows the generator to modulate the latent representation in a class-aware manner, effectively incorporating semantic texture information.

Preliminary experiments evaluated the impact of applying class conditioning not only at the bottleneck but also symmetrically at encoder and decoder layers where Batch Normalization occurs. Visual inspection of the generated spectrograms revealed no improvement in output quality. Conditioning additional decoder layers, especially the third and fourth layers following the bottleneck, often caused a decline in the fidelity of the generated spectrograms. This behavior is likely explained by the presence of skip connections that carry detailed spatial information from the encoder to the decoder, maintaining structural consistency. Adding conditioning in the decoder can disrupt this balance by introducing excessive semantic influence, which interferes with the fine details passed through the skip connections. Therefore, class information is injected only at the bottleneck, where the latent representation is most compressed and semantically rich, enabling effective class-aware modulation without compromising fine-grained details.

**Label Predictor:** A dedicated label predictor is used to provide a class label for conditioning the generator. This module is trained independently prior to the cGAN training, using the same dataset of texture images. The architecture is based on a DenseNet-201 backbone [52], chosen for its strong performance in visual recognition tasks due to its dense connectivity pattern, which facilitates efficient feature reuse and mitigates vanishing gradients in deep networks. To leverage these pre-trained visual features while reducing overfitting on the relatively small texture dataset, the DenseNet-201 backbone is kept frozen during training. The output of the backbone is extracted from the final convolutional block and processed using global average pooling, resulting in a 1920-dimensional feature vector. This vector is then passed to a lightweight classifier head, as illustrated in Figure 1. The classifier consists of a Fully Connected (FC) layer that reduces the dimensionality from 1920 to 256, followed by batch normalization, a LeakyReLU activation with a default negative slope of 0.01, and a final FC layer that maps the 256-dimensional embedding to 9 output logits corresponding to the target classes. Only the parameters of this classifier head are updated during training. To enhance generalization and robustness, extensive data augmentation is applied during the training of the label predictor. This includes scaling the images by factors sampled uniformly from the range [1,1.5], followed by random cropping to a fixed size of 256×256 pixels. Additional augmentations include horizontal and vertical flipping, random rotation, random erasing, and mixup [33]. The label predictor achieves an average classification accuracy of 98.89% on the test set, ensuring reliable label conditioning for the generator during cGAN training.

**Multi-Scale Discriminator:** The discriminator module follows a multi-scale design inspired by pix2pixHD [19], consisting of three independent PatchGAN discriminators [18], each operating on a different resolution of the input. Multi-scale inputs are generated through downsampling blocks with Average Pooling (AvgPool) layers, which reduce spatial resolution by a factor of two at each stage. This setup allows the discriminators to jointly assess visual fidelity at both global and local scales. Each discriminator receives a pair of images: the texture input and either the real or generated spectrogram, both appropriately downsampled (see Figure 1). All discriminators share a common internal architecture consisting of three downsampling layers, followed by two additional convolutional layers that output spatially resolved real/generated predictions. As validated in Section 4.2, a grid search over multiple configurations demonstrated that using three discriminators with three downsampling layers each achieves optimal perceptual performance, based on the LPIPS metric. This multi-scale setup not only enhances perceptual quality but also contributes to more stable adversarial training by capturing texture-spectrogram consistency across varying spatial resolutions. The configuration and parameters of the multi-scale discriminator components are summarized in Table 2.

**Loss Function:** The proposed training objective integrates three loss components: an adversarial loss, a reconstruction loss, and an FM loss. These components collectively optimize both the generator and the multi-scale discriminator, ensuring both realism and fidelity of the generated spectrograms. This design is motivated by the weakly paired nature of the visual-to-tactile translation task, where strict pixel-level correspondence cannot be assumed. While the adversarial loss enforces global realism, the reconstruction loss stabilizes training and preserves coarse temporal–frequency structure, and the FM loss improves perceptual fidelity by aligning intermediate discriminator representations. Empirically, this combination yields more stable training and perceptually meaningful spectrograms compared to using any single loss term alone.

*Adversarial Loss:* The adversarial training is adopted from pix2pix [18] and Pix2PixHD [19], where in our case, the generator *G* learns to produce realistic spectrograms $S_{g}$ from input textures *T*, while the multi-scale discriminator $D={\left\{D_{k}\right\}}_{k=1}^{N_{D}}$ (with $N_{D}=3$) aims to distinguish between real spectrograms $S_{r}$ and generated ones $S_{g}$, conditioned on the same texture input. For each discriminator *k*, the adversarial loss is defined as:

(4) $$L_{\mathrm{GAN}}\left(G,D_{k}\right)=E_{T,S_{r}}\left[\log D_{k}\left(T\|S_{r}\right)\right]+E_{T}\left[\log\left(1-D_{k}\left(T\|S_{g}\right)\right)\right],$$

where T‖S denotes the concatenation of the texture *T* with a spectrogram *S* (either real $S_{r}$ or generated $S_{g}$), forming the input to discriminator $D_{k}$.

*Reconstruction Loss:* To encourage pixel-level similarity between the generated spectrogram and the real one, an $L_{1}$ loss is applied:

(5) $$L_{L1}={∥S_{g}-S_{r}∥}_{1},$$

*Feature Matching Loss:* To further stabilize training and improve perceptual similarity, an FM loss is used. This loss compares intermediate features from all discriminator layers (excluding the final layer) for real and generated spectrograms:

(6) $$L_{\mathrm{FM}}=\frac{1}{N_{D}}\frac{1}{L+1}\sum_{k=1}^{N_{D}}\sum_{l=1}^{L+1}{∥D_{k}^{\left(l\right)}\left(T\|S_{r}\right)-D_{k}^{\left(l\right)}\left(T\|S_{g}\right)∥}_{1},$$

where $D_{k}^{\left(l\right)}$ denotes the *l*-th intermediate feature map from the *k*-th discriminator and L=3 is the number of intermediate downsampling layers per discriminator.

*Objective Function:* The overall training objective is:

(7) $$\min_{G}\max_{D_{1},D_{2},D_{3}}\sum_{k=1}^{N_{D}}L_{\mathrm{GAN}}\left(G,D_{k}\right)+\lambda_{L1}L_{L1}+\lambda_{\mathrm{FM}}L_{\mathrm{FM}},$$

where $\lambda_{L1}=10$ and $\lambda_{FM}=40$ are fixed weights that have been empirically determined to yield good performance. Choosing empirically fixed loss weights is a common practice in cGAN training, as demonstrated by several influential works in the literature, including [17,18,19,35,36,37,53]. These studies similarly select loss weights based on empirical evaluation rather than exhaustive hyperparameter optimization. Given the high computational cost and complexity involved in systematically tuning loss weights, conducting a comprehensive sensitivity analysis is considered an open research problem and is beyond the scope of the present work.

The comparison analysis, detailed in Section 4.3, shows that the hybrid objective effectively balances realism, perceptual similarity, and reconstruction fidelity. This allows the generator to produce accurate and consistent vibrotactile spectrograms that outperform those generated by standard pix2pix [18], pix2pixHD [19], and Residue-Fusion GAN [37] architectures.

### 3.2. Implementation Details

The proposed architecture is implemented using PyTorch-GPU (v1.12.1) [54] on a workstation with an Intel Xeon E5-2640 v3 CPU (2.60 GHz, 8 cores), 16 GB of RAM and an NVIDIA GeForce RTX 2080 Ti GPU. A batch size of 32 is used. Both generator and discriminator of the cGAN are initialized using a normal distribution with a gain of 0.02. The Adam optimizer [55] is used for both, with a learning rate of 0.0002 and betas of (0.5, 0.999), following the settings in pix2pix [18] and Pix2PixHD [19], which demonstrated stable and effective training for similar conditional GAN architectures. A linear learning rate scheduler is employed, gradually reducing the learning rate across the final 100 of the 200 total training epochs, starting at epoch 100. The LPIPS metric is monitored on the validation set during training, and the model with the lowest LPIPS score is selected for evaluation on the test set.

The Label Predictor block is trained for 30 epochs using the Adam optimizer with the same parameters as those described earlier for the cGAN training. The batch size is set to 64. A step-based learning rate scheduler is applied, which halves the learning rate every 5 epochs. Validation accuracy is tracked throughout training, and the model with the best score on the validation set is integrated into the main architecture (see Figure 1).

## 4. Experiments

### 4.1. Dataset and Experimental Setup

**Dataset Selection:** The experiments are conducted using the LMT-108 Surface-Materials dataset [25], which contains visual texture images and corresponding tactile acceleration signals obtained from pen-sliding interactions. Previous studies [13,17,25,37,38] have demonstrated the suitability of this dataset for visual-to-tactile data synthesis. The dataset comprises 108 distinct surface materials grouped into 9 categories. To construct a representative experimental subset, one material is selected from each category, resulting in a total of 9 materials. The selected subset, consistent with the materials presented in [33], includes *Squared Aluminum Mesh*, *Granite Type Veneziano*, *Aluminum Plate*, *Bamboo*, *Solid Rubber Plate*, *Carpet*, *Fine Foam*, *Cardboard*, and *Jeans*. These materials span a broad spectrum of surface properties, supporting a comprehensive evaluation of tactile signal generation across diverse textures. In the experimental setup of the proposed architecture, the training portion of the dataset is used where each material is provided with 10 RGB texture images and the corresponding triaxial acceleration signals (X, Y, Z).

**Visual Data:** Visual data is acquired through texture images that represent the surface geometry and microstructural patterns, which contribute to the resulting vibrotactile sensations. Following previous studies that utilized the LMT-108 dataset for cross-modal learning [37,56], only the non-flash variants of the images are considered to ensure consistent lighting conditions.

To increase the diversity of training inputs and improve generalization, each image undergoes data augmentation ten times. The augmentation pipeline begins by randomly cropping the original 320 × 480 RGB texture images to a resolution of 256 × 256, consistent with the approach in [37]. Additional transformations include random horizontal and vertical flipping, as well as adjustments to image brightness and contrast [37,38]. After augmentation, the RGB images are converted to grayscale, since tactile perception is typically insensitive to color and more reliant on structural cues. Since 10 images per material are used and each undergoes 10 augmentations, the visual dataset comprises 900 grayscale texture images (100 images per class).

**Vibrotactile Data:** To obtain tactile data, only the Z-axis signal is considered among the triaxial acceleration signals (X, Y, Z) available in the dataset, following the approach adopted in previous studies [33,37,38]. This axis is typically associated with the most prominent vibrational characteristics during surface interactions.

Since GANs are primarily designed for 2D data representations, such as images, the raw 1D acceleration signals are transformed into the time–frequency domain using spectrograms. Each Z-axis signal in the dataset spans 4.8 s and is sampled at 10 kHz. A Short-Time Fourier Transform (STFT) is applied using a 512-point Hamming window and a hop size of 128, producing a linear amplitude spectrogram of size 257 × 376, where the magnitude of the complex STFT is used. To enhance data diversity and robustness, each spectrogram is randomly cropped ten times along the temporal axis by extracting 256 consecutive time frames, which correspond to approximately 3.28 s of signal duration. Each cropped spectrogram is resized to 256 × 256 and then transformed into the logarithmic scale. This spectrogram serves as the vibrotactile data, referred to as the Real Spectrogram in Figure 1. Given that 10 vibrotactile signals are used per material and each signal yields 10 randomly cropped spectrograms, the resulting dataset comprises 900 spectrogram images (100 spectrograms per class). The time-domain vibration signals can be reconstructed from the spectrograms using corresponding inverse transformations and the Griffin–Lim algorithm.

**Extended Data:** While data augmentation is commonly used to enhance the variability of visual images, such transformations cannot be directly applied to tactile spectrograms without potentially distorting their temporal and amplitude characteristics. To facilitate the training of the cross-modal generation model, weakly paired data are prepared, following the approach in [37,38]. Starting from 900 texture images and 900 spectrograms, random pairing is repeated ten times within each material category to construct weakly paired samples. This process results in a total of 9000 visual–tactile pairs (1000 per class), where each pairing maintains consistency in material category but does not imply exact instance-level alignment. This approach balances the need for manageable computational complexity with leveraging available data despite the absence of exact instance-level image-to-spectrogram pairs. While this one-to-many pairing introduces ambiguity and may limit the assessment of the model’s full generalizability, it reflects a practical and common compromise in current visual-to-tactile cross-modal research.

All data are normalized to the range [−1,1]. The final dataset of 9000 weakly paired visual–tactile examples was randomly partitioned into training, validation, and test subsets using an 80/10/10 ratio. Stratification was applied at the class level, resulting in 800 training, 100 validation, and 100 test pairs per class. A fixed random seed (random.seed(42)) was used to ensure reproducibility. Care was taken to avoid any overlap of images or spectrograms between subsets, thereby preventing data leakage.

**Evaluation Metric:** To quantitatively evaluate the similarity between real and generated spectrograms, two metrics are employed: the LPIPS [57] and the FID [58]. LPIPS compares deep feature representations extracted from a pretrained neural network, providing a perceptual similarity measure that aligns more closely with human judgment than traditional pixel-wise distances such as $L_{1}$ or $L_{2}$. Although originally proposed for visual image comparison, LPIPS can be extended to evaluate structured 2D data such as spectrograms, which encode rich time–frequency information in a spatial format. This makes it a suitable choice for assessing the perceptual fidelity of generated tactile spectrograms, where minor time–frequency misalignments may not significantly affect perceptual similarity. In this study, both VGG [39] and AlexNet [59] backbones were evaluated for computing LPIPS scores. Empirical observations suggest that AlexNet better reflects human-perceived similarity between spectrograms and is thus adopted as the default backbone in all reported esults.

FID, on the other hand, captures the statistical distance between the distributions of real and generated spectrograms in a deep feature space by comparing their mean and covariance. This global distributional comparison is robust to local misalignments and serves as an effective measure of overall generation quality.

Standard metrics such as PSNR and SSIM are not employed in the evaluation, as they rely on strict pixel-level alignment and assume a one-to-one correspondence between the compared images. In the context of visual-to-tactile translation with weakly paired data, the mapping from a texture input to its corresponding vibrotactile spectrogram is, as mentioned earlier, effectively one-to-many: multiple spectrograms may plausibly represent the same perceptual haptic sensation, differing only in minor temporal or frequency shifts. Such benign misalignments can significantly penalize PSNR or SSIM scores, even when the generated output remains perceptually valid. In contrast, LPIPS and FID are adopted as more suitable alternatives, as they better reflect perceptual similarity and statistical consistency, respectively.

### 4.2. Discriminator Configuration Selection

In order to assess the influence of the number of discriminators and downsampling layers, a grid search is performed using the LPIPS metric, varying both parameters from 1 to 4. For each configuration, 10 independent experiments are conducted to account for random variation, and the reported values represent the mean ± standard deviation. In addition, a two-tailed *t*-test is performed to compare each configuration against the best-performing one, allowing us to determine whether the observed differences are statistically significant.

As shown in Table 3, the optimal configuration, yielding the lowest LPIPS value (0.3113 ± 0.0087), consists of 3 discriminators and 3 downsampling layers. This configuration demonstrates the best perceptual similarity between real and generated spectrograms. For all other configurations, the resulting *p*-values are below the 0.05 threshold, confirming that the performance improvement is statistically significant.

**Figure 2:**
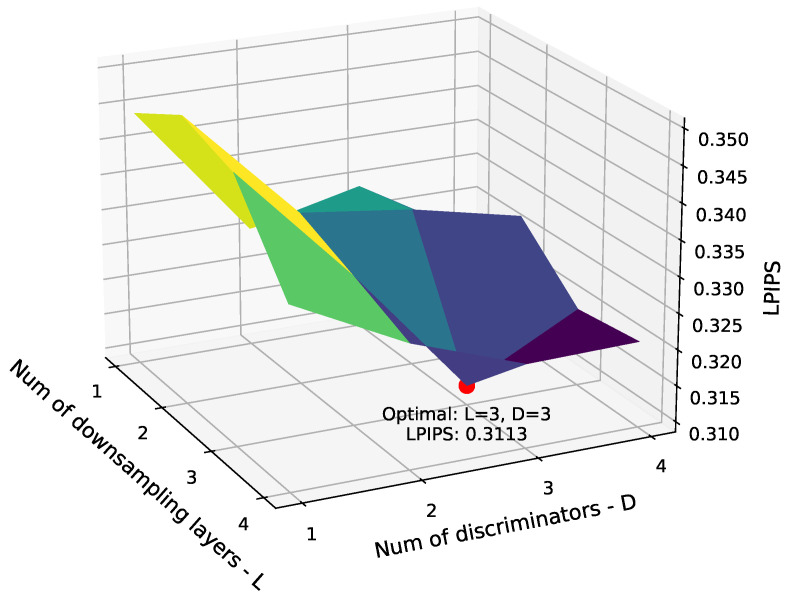
3D plot illustrating the grid search for optimal multi-scale discriminator parameters, including the number of discriminators and downsampling layers selection. The red dot indicates the optimal pair of parameters. Colors in the plot correspond to LPIPS values, with higher values shown in yellow, intermediate in green, and lower in blue.

**Figure 3:**
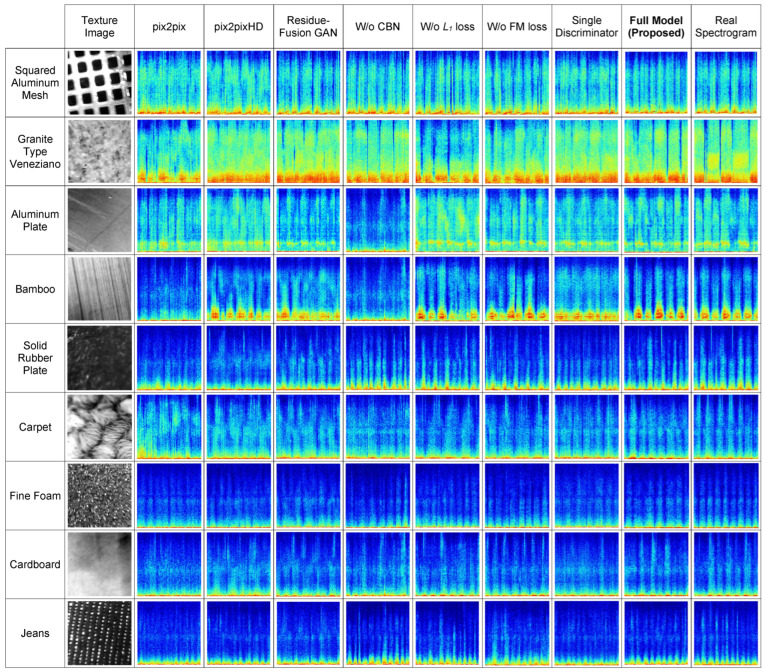
Visual comparison of spectrograms generated by baseline methods, ablation study variants, and the full proposed model, alongside the corresponding real spectrograms, for one representative sample from each of the nine material categories. Colors correspond to the amplitude values in the spectrogram, mapped using the *jet* colormap. It can be observed that the full proposed model synthesizes spectrograms that are visually the closest to the real ones.

For convenience, the LPIPS results from the table are also visualized as a 3D surface in Figure 2, showing the average values as a function of the number of discriminators and downsampling layers. Although LPIPS values differ by less than 0.04 across configurations, visual inspection reveals that even such minor numerical variations can correspond to clearly visible differences in spectrogram quality. This is illustrated in the quantitative comparison presented in Section 4.3, where the configuration with three discriminators (the suggested architecture) is compared against one with a single discriminator (both using three downsampling layers). As shown in Table 4, their LPIPS scores are 0.3113 and 0.3458, respectively, with a difference of only 0.035. Nonetheless, the visual gap in fidelity for the generated spectrograms is evident in Figure 3, particularly for samples from the *Granite Type Veneziano*, *Aluminum Plate*, and *Bamboo* classes, and to a lesser extent for the *Solid Rubber Plate* and *Cardboard* classes.

### 4.3. Comparison Analysis

To comprehensively evaluate the effectiveness of the proposed model, both qualitative and quantitative assessments are conducted. The comparison includes three standard baselines: pix2pix [18], pix2pixHD [19], and Residue-Fusion GAN [37], as well as several ablation variants of the proposed model: Without CBN, Without $L_{1}$ loss, Without FM loss, Single Discriminator (Three downsampling layers), and the Full Model (Proposed).

**Qualitative Evaluation:**Figure 3 shows examples of spectrograms produced by the baseline models, the ablated versions of the proposed method, and the full proposed model, alongside the corresponding real spectrograms, for one representative sample from each of the nine material categories.

Visual differences between generated and real spectrograms are clearly noticeable for several experimental setups. In particular, the spectrograms generated by the baseline methods, pix2pix and pix2pixHD, show more pronounced discrepancies compared to the real ones for the *Squared Aluminum Mesh*, *Granite Type Veneziano*, *Aluminum Plate*, *Bamboo*, *Solid Rubber Plate*, *Carpet*, and *Cardboard* classes. In contrast, the spectrograms generated by the proposed method for these classes are visually much closer to the corresponding real spectrograms.

Furthermore, the necessity of the CBN module becomes particularly evident for classes such as *Aluminum Plate*, *Bamboo*, and *Cardboard*, where its absence leads to significantly degraded spectrogram quality.

Regarding the ablation studies targeting individual loss components, it can be observed that the removal of the $L_{1}$ loss has a stronger negative impact compared to the removal of the FM loss. Poor examples without the $L_{1}$ loss are notable for the *Granite Type Veneziano*, *Aluminum Plate*, and *Carpet* classes. In contrast, the influence of removing the FM loss is most visible in examples from the *Granite Type Veneziano* and *Bamboo* classes, and to a lesser extent for the *Jeans* class.

Concerning the experiments with a single discriminator, as discussed in Section 4.2, substantial visual degradation is observed for the *Granite Type Veneziano*, *Aluminum Plate*, and *Bamboo* classes.

Overall, all evaluated configurations, including the baselines and ablated models, generate spectrograms reasonably close to the real ones for the *Fine Foam* and *Jeans* classes. Notably, these two classes exhibit distinctive fine-grained, point-like structures that strongly differentiate them from the other materials. Such highly distinctive textural characteristics likely make these classes easier to model, contributing to the consistently better generation performance across all methods.

**Quantitative Evaluation:** This section reports the average LPIPS and FID scores along with their standard deviations computed over 10 independent runs for all compared models and ablation variants. As shown in Table 4, the full model (proposed method) consistently achieves the best performance, with the lowest LPIPS score of 0.3113 ± 0.0087 and the lowest FID (38.77 ± 1.92) among all methods. This result highlights the effectiveness of the full model, incorporating all components, including CBN, the $L_{1}$ loss, and FM loss, as well as the use of the proposed discriminator architecture.

As shown in Table 4, the full model (proposed method) consistently achieves the best performance, with the lowest LPIPS score of 0.3113 ± 0.0087 and the lowest FID (38.77 ± 1.92) among all methods. Within the baselines, the Residue-Fusion GAN [37] model performs best, with LPIPS of 0.3198 and FID of 40.76, although still worse than the proposed method.

The pix2pixHD model follows as the next most effective baseline, achieving an LPIPS of 0.3345 and an FID of 43.60, reflecting the advantages of its multi-scale architecture and deeper generator-discriminator design. Notably, pix2pix exhibits the weakest performance, with the highest LPIPS of 0.3513 and FID of 52.23, indicating low perceptual fidelity and poor feature distribution alignment.

The ablation study further highlights the contribution of individual components. Removing CBN results in a noticeable performance drop (LPIPS of 0.3439 and FID of 45.42), underscoring its importance. This indicates that CBN provides effective class conditioning, improving the generator’s ability to produce spectrograms that match the semantic label distribution. Using a single discriminator instead of three also degrades results, with LPIPS increasing to 0.3458 and FID to 47.60. Eliminating the $L_{1}$ or feature matching losses has a smaller impact, yielding LPIPS scores of 0.3278 and 0.3247, and FID values of 41.67 and 41.29, respectively.

In preliminary experiments, training the generator with only the $L_{1}$ loss (without FM loss or/and without CBN) yielded inferior perceptual quality, confirming that the FM loss is necessary to refine fine temporal and frequency structures in the generated spectrograms. All methods show low standard deviations, reflecting stable behavior across runs.

To ensure the reliability of the observed differences, two-sided paired *t*-tests were conducted between the proposed method and each of the other evaluated configurations. In all comparisons, the resulting *p*-values were below the standard threshold of 0.05, indicating that the improvements are statistically significant. Notably, the proposed architecture achieves the best performance and robustness, highlighting the positive impact of combining CBN, $L_{1}$ loss, FM loss, and the multi-scale discriminator configuration.

### 4.4. Impact of Class Conditioning

**Figure 4:**
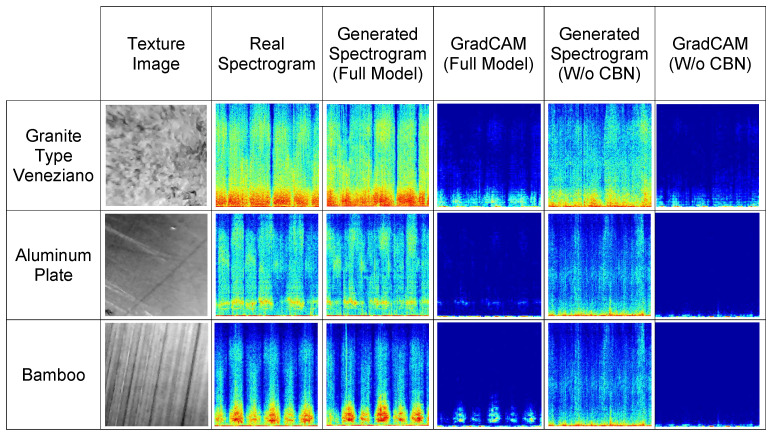
Effect of class conditioning through CBN, visualized with GradCAM. For three representative classes, the texture image, real spectrogram, generated spectrograms (with and without CBN), and their corresponding GradCAM heatmaps are shown. Colors in the spectrograms correspond to amplitude values as in Figure 3, mapped using the *jet* colormap. The model with CBN produces spectrograms that better match real samples and exhibit more focused activations.

Figure 4 shows the effect of label conditioning using CBN, visualized through GradCAM. In the figure, an example is given for each of three classes (*Granite Type Veneziano*, *Aluminum Plate*, and *Bamboo*), where CBN has the most significant impact. It can be observed that the GradCAM heatmaps highlight the regions where the generator focuses most strongly during the synthesis process. These regions often correspond to the most prominent or structurally important parts of the spectrograms, rather than the full structure. In contrast, without CBN, the activations are less focused, and the generated spectrograms deviate more from the real distributions. These observations further demonstrate that CBN plays a crucial role in improving the fidelity and class consistency of the generated outputs.

Regarding the GradCAM implementation, activation maps are extracted from the last transposed convolutional layer in the decoder, which is nearest to the output and encodes high-level features influencing the generated spectrogram. Average pooling over the gradients is used to calculate importance weights, which are combined with activations to produce the GradCAM maps. These heatmaps are normalized to the [0, 1] range using min-max normalization and visualized with a jet colormap overlay.

### 4.5. Failure Cases

**Figure 5:**
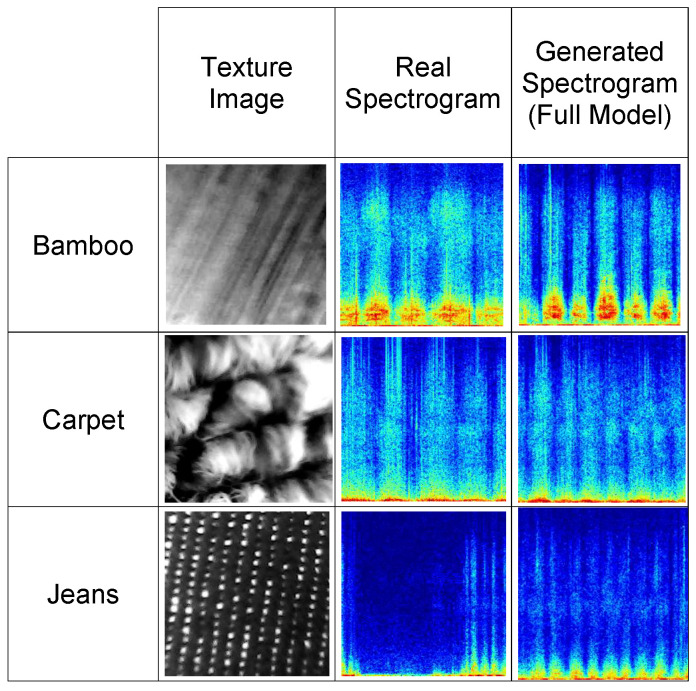
Examples of the most challenging failure cases from three classes. Colors in the spectrograms correspond to amplitude values as in Figure 3, mapped using the *jet* colormap. The generated spectrograms show structural similarities to the real ones but exhibit differences in finer details, such as missing structural features and misplaced high-energy regions. These differences are likely due to weakly paired data and the one-to-many mapping issue.

Although the proposed method demonstrates high performance across most classes, some of the most challenging cases are observed. Figure 5 presents examples from three classes: *Bamboo*, *Carpet*, and *Jeans*. In these cases, the generated spectrograms, although structurally similar to the real ones, exhibit noticeable differences in the finer details. These include missing structural features and misplaced high-energy regions. One potential reason for such deviations is the use of weakly paired data, where the same input texture may correspond to multiple plausible spectrograms within a class. This one-to-many mapping problem challenges the generator to precisely reconstruct a specific target spectrogram, often resulting in outputs that capture the general characteristics but differ from the exact ground truth.

Moreover, subtle variations in texture patterns or class-specific details can further complicate accurate reconstruction. Evaluating these challenging cases highlights the importance of incorporating more discriminative features or attention mechanisms to guide the generation process. Future work could investigate conditioning the generator on more specific instance-level features, which may help reduce ambiguity in the one-to-many mapping and improve the fidelity of generated spectrograms.

## 5. Limitations of the Presented Work

The following are considered limitations of the proposed work:

- **Need for Class-Labeled Data:** The method relies on the availability of well-labeled data, which may not always be feasible in some practical scenarios. To make the method more practical for broader applications, future work could focus on learning from partially labeled or unlabeled data using semi-supervised or domain adaptation techniques.
- **Training with Weakly Paired Data:** The same texture may correspond to multiple spectrograms within a class, creating challenges in precisely reconstructing the target spectrogram. While this results in some imprecision in the generated spectrograms, this isn’t a significant issue. The vibrotactile sensation for materials within a given class should remain similar, allowing a person to experience a consistent tactile sensation when touching the material, even if the exact spectrogram varies.
- **Failure in Fine-Grained Details:** In some cases, the generated spectrograms exhibit deviations from the real ones in finer details, such as missing structural features or misplaced high-energy regions. In future work, expanding the architecture to incorporate bidirectional approaches, such as CycleGAN [20] or DiscoGAN [21], could help address these challenges. While these models are primarily designed for unpaired domain translation and do not exploit paired supervision directly, their cycle-consistency constraints can promote structural fidelity and preserve information that may be lost in single-pass mappings. Integrating such properties into a supervised setting, or combining them with more advanced loss functions, could improve the model’s sensitivity to fine-grained features and lead to more accurate spectrograms.
- **Limited Dataset Scope:** The method has been tested on a dataset with only 9 classes. This limited scope may affect its ability to generalize to other datasets or classes not represented in the current study. Expanding the dataset to include more classes or applying the method to different datasets would help assess the model’s generalization capabilities and identify potential areas for improvement.
- **Absence of Time-Domain Evaluation:** The current approach focuses on spectrogram generation, and while time-domain vibrotactile signals can be reconstructed using algorithms such as Griffin–Lim, this step is not evaluated in the present study. Due to the potential limitations of Griffin–Lim in accurately recovering perceptually faithful vibrations, it remains unclear whether the reconstructed signals would elicit a realistic tactile sensation for end users. A dedicated user study would be necessary in future work to assess the perceptual quality and effectiveness of the reconstructed signals in actual haptic applications.

## 6. Conclusions

This work introduces a class-conditional GAN for converting texture images into vibrotactile spectrograms. By incorporating class-conditional batch normalization (CBN) and utilizing a multi-scale discriminator architecture with three discriminators and three downsampling layers, the proposed model achieves superior perceptual fidelity compared to existing methods such as pix2pix, pix2pixHD, and Residue-Fusion GAN. Ablation studies demonstrate the importance of the key architectural components, while Grad-CAM analysis confirms the role of class conditioning in guiding the generator toward class-relevant spectro-temporal patterns. While the model produces promising results on the LMT-108 dataset, challenges remain in addressing fine-grained discrepancies in the generated spectrograms, including missing structural details or misplaced high-energy regions. Future work could focus on improving generalization through more advanced architectures, refined loss functions, and more effective handling of the one-to-many mapping problem inherent in weakly paired training data. Overall, this research lays the groundwork for vision-driven vibrotactile feedback generation in interactive systems, particularly in virtual material simulation and immersive haptic applications. Beyond these scenarios, the proposed framework may also serve as a complementary perceptual component in broader human–robot interaction and haptic systems, where visual cues can provide additional information about surface properties in the absence of direct tactile sensing. Exploring such integrations within adaptive or closed-loop interaction frameworks represents a promising direction for future research.

## Figures and Tables

**Table 1:** Internal configuration of the generator. Here, K—Kernel size, S—Stride, P—Padding. #Params—Number of parameters. LeakyReLU layers use negative_slope = 0.2.

Component	K	S	P	Output Shape	#Params
Input: Texture + Label	–	–	–	[1, 256, 256] + 9	0
Conv2d	4 × 4	2	1	[64, 128, 128]	1024
LeakyReLU	–	–	–	[64, 128, 128]	0
Conv2d	4 × 4	2	1	[128, 64, 64]	131,072
BatchNorm2d	–	–	–	[128, 64, 64]	256
LeakyReLU	–	–	–	[128, 64, 64]	0
Conv2d	4 × 4	2	1	[256, 32, 32]	524,288
BatchNorm2d	–	–	–	[256, 32, 32]	512
LeakyReLU	–	–	–	[256, 32, 32]	0
Conv2d	4 × 4	2	1	[512, 16, 16]	2,097,152
BatchNorm2d	–	–	–	[512, 16, 16]	1024
LeakyReLU	–	–	–	[512, 16, 16]	0
Conv2d	4 × 4	2	1	[512, 8, 8]	4,194,304
BatchNorm2d	–	–	–	[512, 8, 8]	1024
LeakyReLU	–	–	–	[512, 8, 8]	0
Conv2d	4 × 4	2	1	[512, 4, 4]	4,194,304
BatchNorm2d	–	–	–	[512, 4, 4]	1024
LeakyReLU	–	–	–	[512, 4, 4]	0
Conv2d	4 × 4	2	1	[512, 2, 2]	4,194,304
BatchNorm2d	–	–	–	[512, 2, 2]	1024
LeakyReLU	–	–	–	[512, 2, 2]	0
Conv2d	4 × 4	2	1	[512, 1, 1]	4,194,304
CBN	–	–	–	[512, 1, 1]	10,240
ReLU	–	–	–	[512, 1, 1]	0
ConvTranspose2d	4 × 4	2	1	[512, 2, 2]	4,194,304
BatchNorm2d	–	–	–	[512, 2, 2]	1024
ReLU	–	–	–	[1024, 2, 2]	0
ConvTranspose2d	4 × 4	2	1	[512, 4, 4]	8,388,608
BatchNorm2d	–	–	–	[512, 4, 4]	1024
ReLU	–	–	–	[1024, 4, 4]	0
ConvTranspose2d	4 × 4	2	1	[512, 8, 8]	8,388,608
BatchNorm2d	–	–	–	[512, 8, 8]	1024
ReLU	–	–	–	[1024, 8, 8]	0
ConvTranspose2d	4 × 4	2	1	[512, 16, 16]	8,388,608
BatchNorm2d	–	–	–	[512, 16, 16]	1024
ReLU	–	–	–	[1024, 16, 16]	0
ConvTranspose2d	4 × 4	2	1	[256, 32, 32]	4,194,304
BatchNorm2d	–	–	–	[256, 32, 32]	512
ReLU	–	–	–	[512, 32, 32]	0
ConvTranspose2d	4 × 4	2	1	[128, 64, 64]	1,048,576
BatchNorm2d	–	–	–	[128, 64, 64]	256
ReLU	–	–	–	[256, 64, 64]	0
ConvTranspose2d	4 × 4	2	1	[64, 128, 128]	262,144
BatchNorm2d	–	–	–	[64, 128, 128]	128
ReLU	–	–	–	[1, 256, 256]	0
ConvTranspose2d	4 × 4	2	1	[1, 256, 256]	2049
Output: Tanh	–	–	–	[1, 256, 256]	0
Total Trainable Parameters					54,418,049

**Table 2:** Internal configuration of the discriminator. Here, K—Kernel size, S—Stride, P—Padding. #Params—Number of parameters. LeakyReLU layers use negative_slope = 0.2.

Component	K	S	P	Output Shape	#Params
Input: Texture + Spectrograml	–	–	–	[1, 256, 256] + [1, 256, 256]	0
Conv2d	4 × 4	2	2	[64, 129, 129]	2112
LeakyReLU	–	–	–	[64, 129, 129]	0
Conv2d	4 × 4	2	2	[128, 65, 65]	131,200
BatchNorm2d	–	–	–	[128, 65, 65]	256
LeakyReLU	–	–	–	[128, 65, 65]	0
Conv2d	4 × 4	2	2	[256, 33, 33]	524,544
BatchNorm2d	–	–	–	[256, 33, 33]	512
LeakyReLU	–	–	–	[256, 33, 33]	0
Conv2d	4 × 4	1	2	[512, 34, 34]	2,097,664
BatchNorm2d	–	–	–	[512, 34, 34]	1024
LeakyReLU	–	–	–	[512, 34, 34]	0
Conv2d	4 × 4	1	2	[1, 35, 35]	8193
AvgPool2d	3 × 3	2	1	[1, 1, 128, 128]	0
Conv2d	4 × 4	2	2	[64, 65, 65]	2112
LeakyReLU	–	–	–	[64, 65, 65]	0
Conv2d	4 × 4	2	2	[128, 33, 33]	131,200
BatchNorm2d	–	–	–	[128, 33, 33]	256
LeakyReLU	–	–	–	[128, 33, 33]	0
Conv2d	4 × 4	2	2	[256, 17, 17]	524,544
BatchNorm2d	–	–	–	[256, 17, 17]	512
LeakyReLU	–	–	–	[256, 17, 17]	0
Conv2d	4 × 4	1	2	[512, 18, 18]	2,097,664
BatchNorm2d	–	–	–	[512, 18, 18]	1024
LeakyReLU	–	–	–	[512, 18, 18]	0
Conv2d	4 × 4	1	2	[1, 19, 19]	8193
AvgPool2d	3 × 3	2	1	[1, 1, 64, 64]	0
Conv2d	4 × 4	2	2	[64, 33, 33]	2112
LeakyReLU	–	–	–	[64, 33, 33]	0
Conv2d	4 × 4	2	2	[128, 17, 17]	131,200
BatchNorm2d	–	–	–	[128, 17, 17]	256
LeakyReLU	–	–	–	[128, 17, 17]	0
Conv2d	4 × 4	2	2	[256, 9, 9]	524,544
BatchNorm2d	–	–	–	[256, 9, 9]	512
LeakyReLU	–	–	–	[256, 9, 9]	0
Conv2d	4 × 4	1	2	[512, 10, 10]	2,097,664
BatchNorm2d	–	–	–	[512, 10, 10]	1024
LeakyReLU	–	–	–	[512, 10, 10]	0
Conv2d	4 × 4	1	2	[1, 11, 11]	8193
Total Trainable Parameters					8,296,515

**Table 3:** LPIPS scores depending on the number of discriminators and downsampling layers. Values are obtained during grid search, averaged over 10 runs, and reported with standard deviation (mean ± std). The optimal LPIPS value is highlighted in bold.

		Num of Downsampling Layers (L)			
		**1**	**2**	**3**	**4**
Num of discriminators (D)	1	0.3442 ± 0.0106	0.3486 ± 0.0099	0.3458 ± 0.0080	0.3337 ± 0.0084
	2	0.3254 ± 0.0078	0.3328 ± 0.0090	0.3294 ± 0.0084	0.3258 ± 0.0100
	3	0.3291 ± 0.0102	0.3308 ± 0.0096	**0.3113 ± 0.0087**	0.3202 ± 0.0073
	4	0.3200 ± 0.0079	0.3274 ± 0.0072	0.3194 ± 0.0057	0.3205 ± 0.0101

**Table 4:** LPIPS and FID scores (lower is better) for all evaluated variants on LMT-108 samples. The proposed method achieves the best average performance (highlighted in bold). Values are averaged over 10 runs and reported with standard deviation (mean ± std).

Method	LPIPS	FID
pix2pix	0.3513 ± 0.0089	52.23 ± 2.01
pix2pixHD	0.3345 ± 0.0105	43.60 ± 1.88
Residue-Fusion GAN	0.3198 ± 0.0075	40.76 ± 1.83
W/o CBN	0.3439 ± 0.0093	45.42 ± 2.10
W/o L_1_ loss	0.3278 ± 0.0087	41.67 ± 2.14
W/o FM loss	0.3247 ± 0.0068	41.29 ± 1.98
Single Discriminator	0.3458 ± 0.0080	47.60 ± 1.91
**Full Model (Proposed)**	**0.3113 ± 0.0087**	**38.77 ± 1.92**

## Data Availability

Data sharing is not applicable to this article.

## Abbreviations

The following abbreviations are used in this manuscript:

AR	Augmented Reality
AvgPool	Average Pooling
CBN	Conditional Batch Normalization
cGAN	conditional Generative Adversarial Network
CM-AVAE	Cross-Modal Adversarial Variational Autoencoder
DE	SPatially-Adaptive (DE) normalization
FC	Fully Connected
FID	Fréchet Inception Distance
FM	Feature Matching
GAN	Generative Adversarial Network
LPIPS	Learned Perceptual Image Patch Similarity
LP	Short-Time Fourier Transform (STFT)
SPADE	SPatially-Adaptive (DE) normalization
STFT	Short-Time Fourier Transform
VAEs	Variational Autoencoders
VR	Virtual Reality
WGAN-GP	Wasserstein Generative Adversarial Network with Gradient Penalty
